# Oxidation Performance of Nano-Layered (AlTiZrHfTa)N*_x_*/SiN*_x_* Coatings Deposited by Reactive Magnetron Sputtering

**DOI:** 10.3390/ma17122799

**Published:** 2024-06-07

**Authors:** Djallel Eddine Touaibia, Sofiane Achache, Abdelhakim Bouissil, Fabrice Parent, Jaafar Ghanbaja, Alina Gorbunova, Pavel S. Postnikov, Mohamed Mehdi Chehimi, Frederic Schuster, Frederic Sanchette, Mohamed El Garah

**Affiliations:** 1 LASMIS—Laboratory of Mechanical & Materials Engineering, Antenne de Nogent-52, Pôle Technologique de Sud-Champagne, 52800 Nogent, France; soufyane.achache@utt.fr (S.A.); abdelhakim.bouissil@utt.fr (A.B.); fabrice.parent@utt.fr (F.P.); frederic.sanchette@utt.fr (F.S.); 2 LRC CEA-LASMIS, Nogent International Centre for Coating Innovation (NICCI), Pôle Technologique de Sud-Champagne, 52800 Nogent, France; 3 Institut Jean Lamour (UMR CRS 7198), Université de Lorraine, 54000 Nancy, France; jaafar.ghanbaja@univ-lorraine.fr; 4 Research School of Chemistry and Applied Biomedical Sciences, Tomsk Polytechnic University, Tomsk 634050, Russia; aag84@tpu.ru (A.G.); pavelpostnikov@gmail.com (P.S.P.); 5 ITODYS, CNRS, UMR 7086, University of Paris, 15 rue JA de Baïf, 75013 Paris, France; mmchehimi@yahoo.fr; 6 Commissariat à l’Energie Atomique et aux énergies Alternatives (CEA) Saclay, 91191 Gif-sur Yvette, France; frederic.schuster@cea.fr

**Keywords:** high-entropy alloys, coatings, magnetron sputtering, (AlTiZrHfTa)/SiN*_x_*, oxidation, nano-layered

## Abstract

This work uses the direct current magnetron sputtering (DCMS) of equi-atomic (AlTiZrHfTa) and Si targets in dynamic sweep mode to deposit nano-layered (AlTiZrHfTa)N*_x_*/SiN*_x_* refractory high-entropy coatings (RHECs). Transmission electron microscopy (TEM), field emission scanning electron microscopy (FESEM), thermogravimetric analysis (TGA), X-ray diffraction (XRD), and X-ray photoelectron spectroscopy (XPS) are used to investigate the effect of Si addition on the oxidation behavior of the nano-layered coatings. The Si-free nitride coating exhibits FCC structure and columnar morphology, while the Si-doped nitride coatings present a FCC (AlTiZrHfTa)N/amorphous-SiN*_x_* nano-layered architecture. The hardness decreases from 24.3 ± 1.0 GPa to 17.5 ± 1.0 GPa because of the nano-layered architecture, whilst Young’s modulus reduces from 188.0 ± 1.0 GPa to roughly 162.4 ± 1.0 GPa. By increasing the thickness of the SiN*_x_* nano-layer, k_p_ values decrease significantly from 3.36 × 10^−8^ g^2^ cm^−4^ h^−1^ to 6.06 × 10^−9^ g^2^ cm^−4^ h^−1^. The activation energy increases from 90.8 kJ·mol^−1^ for (AlTiZrHfTa)N*_x_* nitride coating to 126.52 kJ·mol^−1^ for the (AlTiZrHfTa)N*_x_*/SiN*_x_* nano-layered coating. The formation of a FCC (AlTiZrHfTa)-N*_x_*/a-SiN*_x_* nano-layered architecture results in the improvement of the resistance to oxidation at high temperature.

## 1. Introduction

The expanding strives for innovative materials—required to operate in demanding thermal and mechanical environments in industry—leads to several initiatives in academia and industry. The most crucial characteristics needed for protective coatings are high hardness, strong adherence to surfaces, high strength at high temperatures, and good oxidation resistance [1,2]. This is why innovative coatings with increased hardness, wear, and oxidation resistance are urgently required and should be studied. Binary system coatings such as TiN and CrN [3], ternary coatings such as CrAlN [4,5], multilayer coatings such as TiC/VC [6], TiAlN/CrN [7], TiN/Si_3_N_4_ [8], and other multilayer nano-composite coatings [9,10,11] have all been steadily investigated and used extensively. Multilayers showed interesting properties in terms of hardness, wear, and oxidation resistances [12,13,14,15,16].

The alloying concept of high-entropy alloys (HEAs), or multi-principal-element alloys, is believed to hold promise in this regard. Generally, HEAs are formed of at least five elements with atomic ratios varying from 5 to 35 at.%, characterized by a single-phased solid solution [17,18]. They have a high mixing entropy compared to conventional alloys in the solution state, allowing them to form stable solid solutions at high temperatures and inhibiting the formation of undesired brittle intermetallic compounds [19,20,21]. Hence, HEAs are considered as a potential class of materials for protective coatings [22,23,24,25].

Refractory high-entropy alloys (RHEAs) based on transition group metals have interesting applications in several devices [8,26,27]. However, they frequently exhibit room-temperature brittleness and poor oxidation resistance at elevated temperatures [28,29,30,31]. Several studies have been undertaken in the past to improve the oxidation resistance of refractory alloys through alloying additions containing metals such as Al, Cr, and Si. When introduced in an appropriate amount, these elements may stimulate the formation of protective oxide top-layers like: Al_2_O_3_, Cr_2_O_3_, and SiO_2_ [29,32,33,34].

The critical challenge for RHEAs is still high-temperature oxidation resistance. Muller et al. [35] enhanced the protective properties of TaMoCrTiAl RHEAs by alloying with Cr and Al. The combination of Al and Cr resulted in the development of adhering and protective CrTaO_4_ oxide scale. The CrTaO_4_ layer was formed in the 500–1200 °C temperature range. Sheikh et al. [29] performed an aluminizing process on the ductile Al_0.5_Cr_0.5_Nb_0.5_Ta_0.5_Ti_0.5_. They reported an improvement in the oxidation resistance of the coating at 800 °C.

According to the literature, adding silicon increases oxidation resistance at high temperatures [24,36]. Yu et al. [24] used magnetron sputtering technology to deposit (AlCrTiZrMo)-Si_x_-N coatings with varying silicon contents and investigated its effect on the structure and properties of the coatings. The addition of silicon resulted in grain refinement of the microstructure due to the formation of nano-composite architecture (FCC (AlCrTiZrMo)N nano-crystallites encapsulated by the amorphous Si_3_N_4_ phases). Due to the increase in Si content in the coating, high hardness, and Young’s modulus were achieved at 28.5 GPa and 325.4 GPa, respectively. When the Si content exceeds 4.5 at.%, the hardness and Young’s modulus decreased, due to an excess of amorphous boundary phase in the coating.

Different multilayered coatings, such as (TiZrNbTaHf)N/MoN [37], (TiZrNbTaHf)N/WN [38] prepared by vacuum-arc deposition, AlCrRuTaTiZr/(AlCrRuTaTiZr)N [25] and AlCrMoNbZr/(AlCrMoNbZr)N [39,40], deposited by reactive magnetron sputtering, were investigated. They exhibit good corrosion resistance, interface stability and mechanical properties. These multi-layer high-entropy nitride ceramic coatings (HENCFs) offer tunable features and promise for use in tool coating materials [41].

The aim of this study is to depict the Si and the nano-layered architecture effects on the oxidation resistance enhancement of the (AlTiZrHfTa)N*_x_* RHEC, obtained at R_N2_ = 10% with (R_N2_ = N_2_/(Ar + N_2_)). In fact, this work is performed in line with the previous study with a deep focus on (AlTiZrHfTa)N*_x_* RHECs and the improvement of their oxidation resistance [42]. The FCC (AlTiZrHfTa)N*_x_*/a-SiN*_x_* nano-layered coatings were deposited by reactive DCMS of equi-atomic (AlTiZrHfTa) and Si targets in dynamic sweep mode, to provide adjustable coating characteristics [43]. Furthermore, the microstructure evolution, the chemical composition, the structure, and the mechanical properties are also addressed.

## 2. Materials and Methods

### 2.1. Deposition of the Coatings

The FCC (AlTiZrHfTa)N*_x_*/a-SiN*_x_* RHECs were deposited by means of DP 650 Alliance Concept device (. The RHECs were deposited on different substrates: flat glass for X-ray diffraction (XRD) analysis, on Si (100) for scanning electron microscopy (SEM), electron probe micro-analysis (EPMA), and transmission electron microscopy (TEM) investigations, and (0 0 0 1)-oriented sapphires substrates for TGA. The co-deposition was carried out by reactive DCMS of 99.99% pure equi-atomic (AlTiZrHfTa) HEA and 99.99% (brazed) Si targets. Prior to loading into the reactor, the substrates were ultrasonically cleaned in acetone and ethanol. The distance between the targets and the substrate holder was 6 cm.

**Figure 1:**
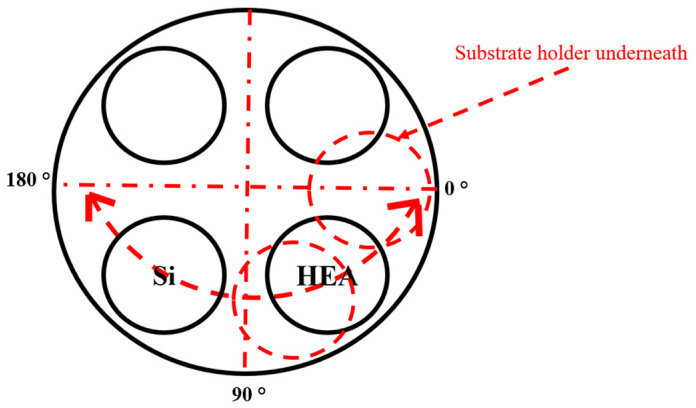
Targets disposition inside the reactor.

Before deposition, the targets were homogeneously sputtered by argon ions for 10 min at (1 Pa). Afterwards, the substrates’ surfaces were etched with argon ions (1 Pa) by RF power of 200 watts for a during 23 min. The HEA target current intensity was 1.0 A; however, the current intensity of Si target (I_Si_) varied from 0 A to 0.4 A. The coatings were deposited, at 1 Pa, under R_N2_ flow ratios of 10%. During deposition, a rotating substrate holder was used (rotating speed = 2 rpm), with a sweep mode (amplitude = 180 °), Figure 1. The deposition durations were adjusted to obtain at least 2 µm-thickness for all coatings.

### 2.2. Sample Analysis

#### 2.2.1. Structure and Microstructure Characterization

The crystal phase identification was carried out by X-ray diffraction on the D8-Advance Bruker diffractometer (Bruker, Billerica, MA, USA) in Bragg–Brentano symmetrical mode, with a radiation source of Cu Kα (λ = 1.544184 Å, 40 kV, 40 mA), a scanning range of 20 ° to 100 ° with a step speed of 0.02 °/s. TEM studies of the coatings were carried out by using a JEM-ARM 200F cold Field Emission Gun (FEG) (JEOL, Tokyo, Japan) (TEM/Scanning Transmission Electron Microscopy STEM). The TEM instrument was running at 200 kV and equipped with an image corrector and a spherical aberration (Cs) probe (point resolution 0.12 nm in TEM mode and 0.078 nm in STEM mode). Focused Ion Beam/Scanning Electron Microscopy (FIB/SEM) FEI Helios NanoLab 600i (FEI, Hillsboro, OR, USA) with platinum Gas Injection System was used to prepare TEM samples. The chemical compositions of the (AlTiZrHfTa)N*_x_*/a-SiN*_x_* RHECs were analyzed using EPMA (microprobe JEOL JXA-8530F, JEOL, Tokyo, Japan). The bonding structure of the nano-layered coatings was characterized by X-ray photoelectron spectroscopy using an NEXSA apparatus (Thermo, East Grinsted, UK) fitted with a monochromatic X-ray Al Kα source (energy = 1486.6 eV and power = 150 W).

A TESCAN MIRA Field emission source electron–Schottky electron gun was used to measure the thickness and examine the morphology of the coatings’ cross-sections and surfaces.

#### 2.2.2. Mechanical Properties

The deposited high-entropy coatings’ nano-hardness (H) and reduced Young’s modulus (ER) were measured by using a HYSITRON, TI980 Triboindenter instrument (Bruker Nano, Inc, Eden Prairie, MN, USA) equipped with a Berkovitch indenter (Bruker Nano, Inc, Eden Prairie, MN, USA). To eliminate the effects of substrate stiffness, the maximum penetration depth is set to less than 10% of the coating thickness. The values of hardness and Young’s modulus were calculated by taking an average of thirty indents.

#### 2.2.3. High-Temperature Oxidation Tests

A thermogravimetric analyzer (TGA, SETARAM, SETSYS evolution, (SETARAM Instrumentation KEP Technologies, Caluire-et-Cuire, France) was used to conduct the oxidation tests in a dry-air (80% N_2_, 20% O_2_) atmosphere. The (0 0 0 1)-oriented sapphires were dual-side coated and served as the test specimens for the TGA. Two different testing protocols were utilized: the first was dynamic and the second protocol was static; more details are provided in reference [42]. Both protocols are used to evaluate the oxidation resistance of the coatings and assess the effect of Si addition on the oxidation performances of the RHECs.

## 3. Results and Discussion

### 3.1. Microstructure of (AlTiZrHfTa)N_x_/SiN_x_ Thin Coatings

**Figure 2:**
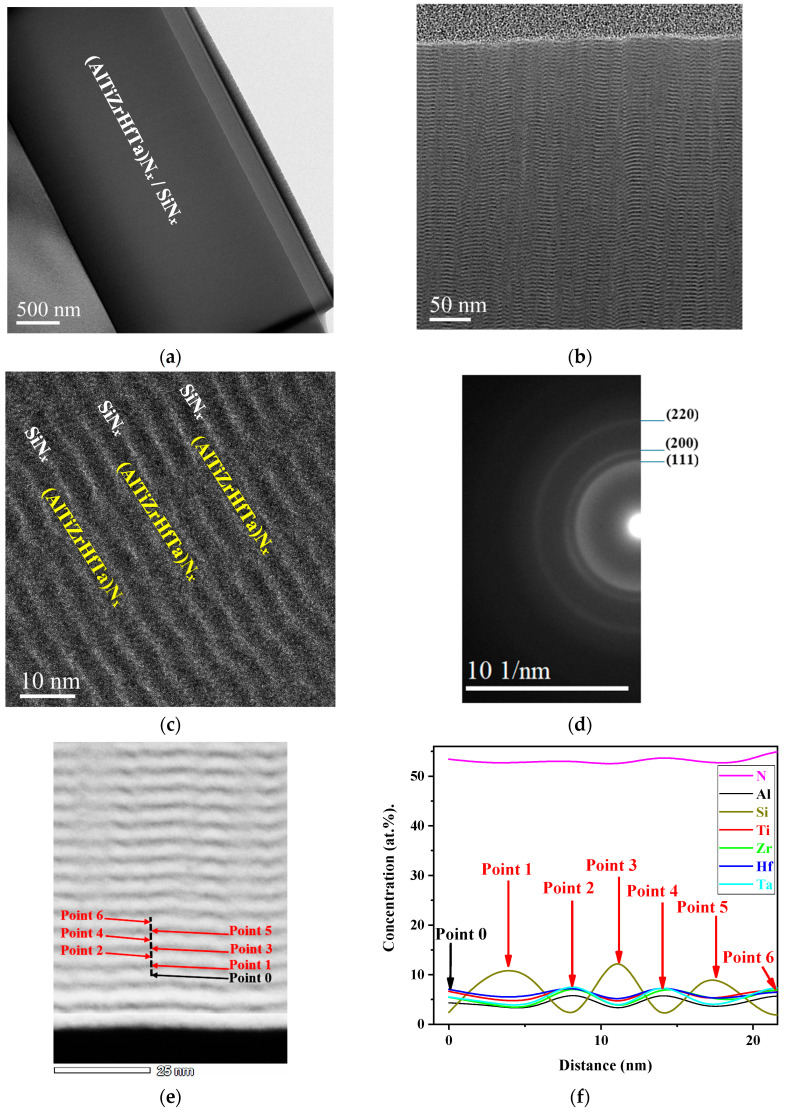
(**a**) Bright filed micrograph of the (AlTiZrHfTa)N*_x_* coating at R_N2_ = 10% obtained for I_Si_ = 0.2 A. (**b**) Zoom-in on nano-layered FCC (AlTiZrHfTa)N*_x_*/a-SiN*_x_*. (**c**) HRTEM micrograph. (**d**) SAED pattern of the FCC (AlTiZrHfTa)N*_x_*/SiN*_x_* coating obtained for I_Si_ = 0.2 A. (**e**) STEM HAADF image of the FCC (AlTiZrHfTa)N*_x_*/SiN*_x_* coating obtained for I_Si_ = 0.2 A, showing the scan line of nano-probe EDX and (**f**) compositional profiles across the nano-layered thin coating.

TEM investigations were performed on the selected samples of (AlTiZrHfTa)N*_x_* nitride coating and high-entropy nitride coating obtained for I_Si_ = 0.2 A. This latter coating presented a protective one of the best oxidation behavior (Section 3.6.2). Figure 2 presents the cross-sectional TEM bright field micrographs, and the selected area presents electron diffraction (SAED) patterns of the FCC (AlTiZrHfTa)N*_x_*/amorphous SiN*_x_* obtained for I_Si_ = 0.2 A. The HRTEM (high-resolution transmission electron microscopy) micrograph and compositional profile of (AlTiZrHfTa)N*_x_* RHECs obtained for I_Si_ = 0.2 A are also shown.

In the previous study [42], the results showed that the (AlTiZrHfTa)N*_x_* nitride coating exhibits a stable FCC-single phased structure. In addition, the nitride coating features a coarse, fiber-like grain structure in the coating growth, as well as a V-shaped growth of faceted columns, indicating T zone pattern growth (Barna Model) [44]. (AlTiZrHfTa)N*_x_* nitride presented a monolithic architecture.

When (AlTiZrHfTa)N*_x_*/SiN*_x_* is deposited, the coating is denser (Figure 2a) compared to the (AlTiZrHfTa)N*_x_* nitride coating, which has a columnar morphology [42]. For (AlTiZrHfTa)N*_x_*/SiN*_x_* coating, obtained at I_Si_ = 0.2 A, an obvious nano-layered structure was observed with clear interfaces between the Si–N nano-layer and (AlTiZrHfTa)N*_x_* nano-layer (Figure 2b,c). The period α = t (AlTiZrHfTa)N*_x_* + t SiN*_x_* layer, where t (AlTiZrHfTa)N*_x_* and t SiN*_x_* are the thicknesses of the (AlTiZrHfTa)-N*_x_* nano-layer and the SiN*_x_* nano-layer, was measured at around 5 nm with t_(AlTiZrHfTa)N_ layer = 3.5 nm, and t_SiN*x*_ layer = 1.5 nm (Figure 2c). A similar configuration was observed by Cai et al. [45] and Xu et al. [46] when investigating dual phase CoCrCuFeNi/Al nano-layered and TiAlN/TiN, TiAlN/ZrN nano-layered coatings, respectively. In our case, the nano-layered architecture is formed with a thinner period. During the process, the small target–substrate distance (6 cm) and a low sweep rate (2 rpm) made the substrates become exposed separately, for a certain duration, to each target.

The HRTEM image shows the presence of a nano-layered structure (Figure 2c). The SAED pattern, presented in Figure 2d, reveals that the SiN*_x_* nano-layer is amorphous and the (AlTiZrHfTa)N*_x_* nano-layer is a clear FCC crystalline structure (Figure 2d). Furthermore, line-scan EDS profiles, illustrated in Figure 2e,f indicate clearly the presence of fluctuations in Si and (Al,Ti,Zr,Hf,Ta) concentrations between neighboring nano-layers, as well as a relatively stable N concentration profile. These indicates that (1) all elements are bounded with nitrogen and (2) the multilayer exists at nano-metric scale ((AlTiZrHfTa)N*_x_* + SiN*_x_*).

**Figure 3:**
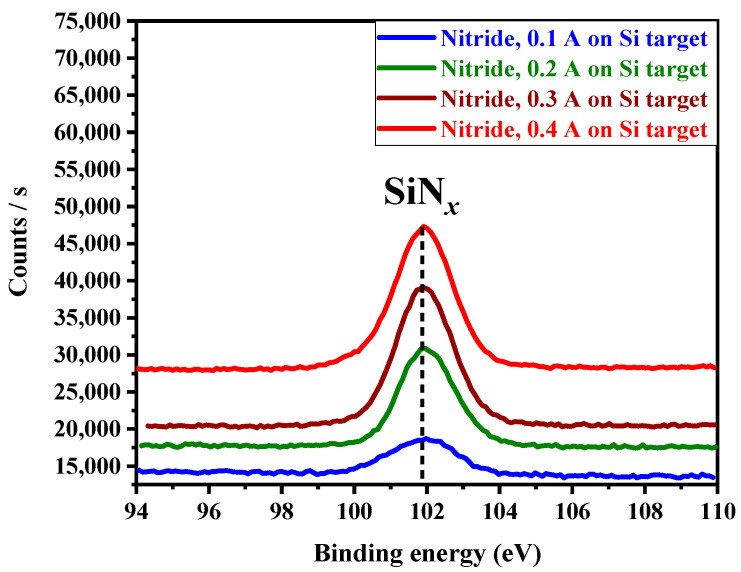
Si 2p XPS spectrum of (AlTiZrHfTa)-N*_x_*/SiN*_x_* nitride coatings as a function of I_Si_.

The XPS technology has been used to identify the different bounding between the constituent elements of the RHECs. Our group has published the XPS spectra, where the full spectrum was shown, of the (AlTiZrHfTa)N*_x_* coating for various nitrogen flow rates (R_N2_) [47,48]. In this work, we proceeded with the silicon bounding only and the results are presented in Figure 3. The Si 2p spectrum shows the presence of one peak at 101.8 eV that can be assigned to Si–N bonds in SiN*_x_*. Similar trend have been reported by Shi et al. [49] and Yu et al. [24], revealing the formation of a Si_3_N_4_ component. As the I_Si_ increases, the thickness of the SiN*_x_* layer increases.

### 3.2. Structure and Microstructure

**Figure 4:**
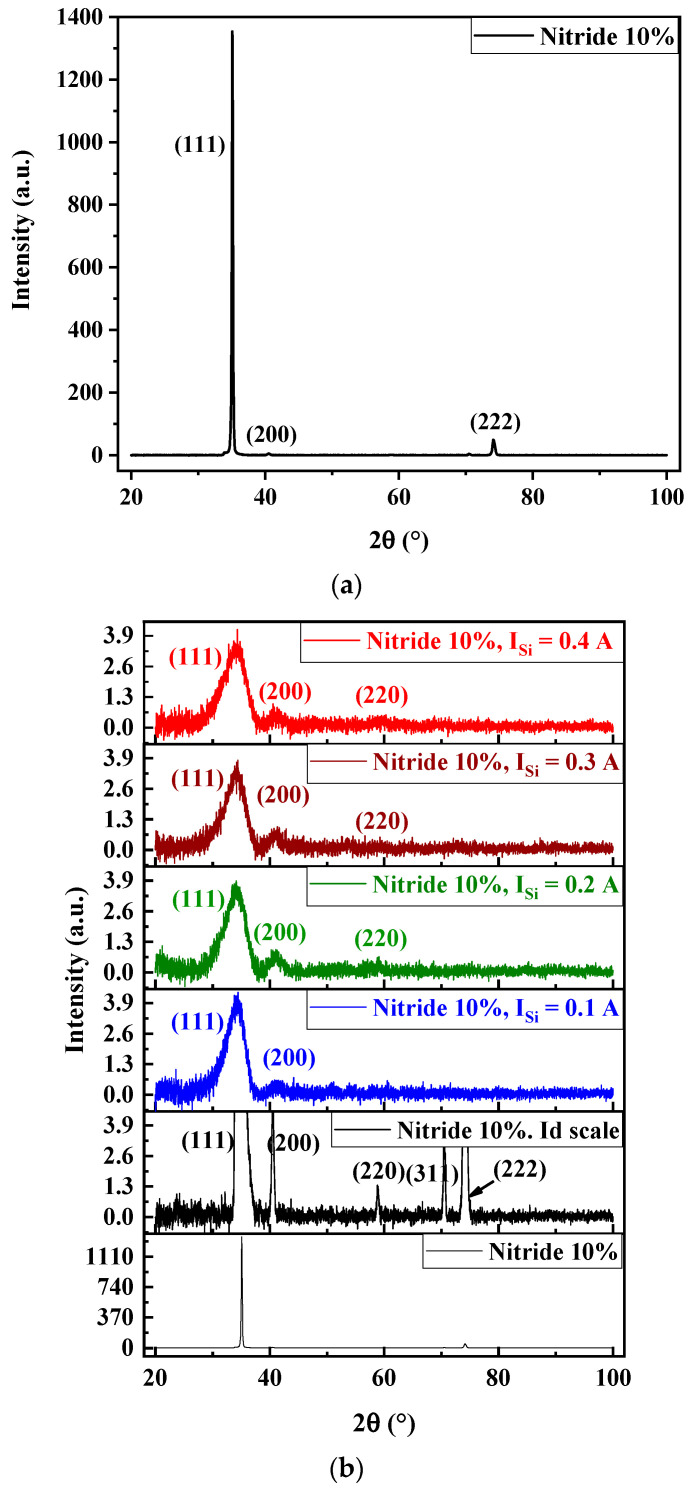
X-ray diffraction patterns of (**a**) Si-free (AlTiZrHfTa)N*_x_* nitride coating and (**b**) FCC (AlTiZrHfTa)N*_x_*/a-SiN*_x_* coatings as a function of I_Si_.

Figure 4 illustrates the XRD patterns of FCC (AlTiZrHfTa)N*_x_*/a-SiN*_x_* RHECs as a function of I_Si_, for R_N2_ = 10%. The diffraction peaks were identified to TaN structure (CIF No. 2310957). The (AlTiZrHfTa)N*_x_* (R_N2_ = 10%) nitride coating exhibits single-phased [NaCl-type (β1)] FCC solid solution structure (Figure 4a), rather than any complex phase separations [50,51,52]. This trend has been figured out in the previous studies [42,48] and observed in the literature as well, for several high-entropy thin coatings like: AlCrTaTiZr [52], AlCrMoSiTi [53], TiVCrZrHf [54], and TiTaZrHfW [55].

When (AlTiZrHfTa)N*_x_*/SiN*_x_* coatings are deposited, XRD patterns (Figure 4b) show low-intensity and large diffraction peaks (Figure 4b), compared to that of nitride, which is directly linked to the formation the nano-layered architecture, alternating FCC (AlTiZrHfTa)N*_x_* nano-layers and amorphous SiN*_x_* nano-layers as shown in (Section 3.1). In fact, the SiN*_x_*-based compound presents an amorphous aspect [8,50,56,57] and inhibits the growth of the nitride columns [8,58], resulting in the broadening of the peaks when depositing (AlTiZrHfTa)N*_x_*/SiN*_x_*.

The average grain size (Ø) is calculated from the most intense (111) peaks, by using the Scherrer equation [59]. The mean grain size values decreased from 45.92 nm for FCC (AlTiZrHfTa)-N*_x_* coating to ~2 nm for all the FCC (AlTiZrHfTa)-N*_x_*/a-SiN*_x_* coatings (Table 1).

However, according to the comprehensive research so far, at room temperature, refractory metal nitrides deposited on substrates require some time to achieve a high-crystalline structure [42,54,55,60]. Nieborek et al. [61] observed that the grain size of the magnetron sputtered TiN increases as the coating grows. They clearly showed (Figure 7d in reference [61]) that at the interface between TiN film and the substrate, the coating is almost amorphous. The grains with small size (almost amorphous) can be found at the interface with the substrate, and as the coating becomes thicker, the grain size increases, reaching its maximum near the surface. In this study, the deposition of SiN*_x_* resumes crystallization and renders all layers nearly amorphous. That is why there is a direct drop in the average grain size after the introduction of the SiN*_x_* layer and no dependence on the SiNx layer thickness (Table 1). The further increase in I_Si_ could lead to the increase in the deposition rate, which leads to the increase in the thickness of the SiN*_x_* nano-layer.

### 3.3. Morphology of FCC (AlTiZrHfTa)N_x_/a-SiN_x_ Thin Coatings

**Figure 5:**
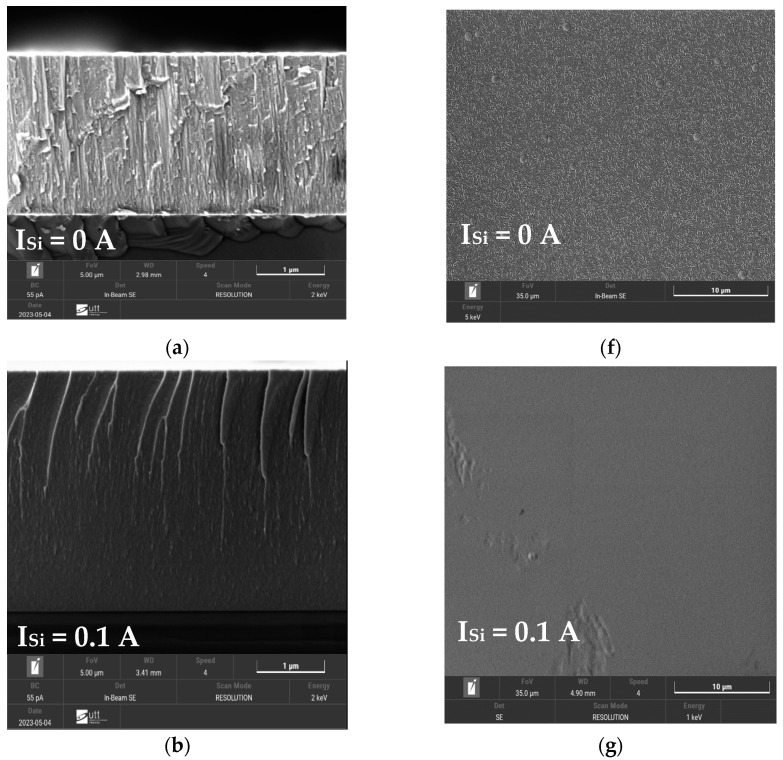

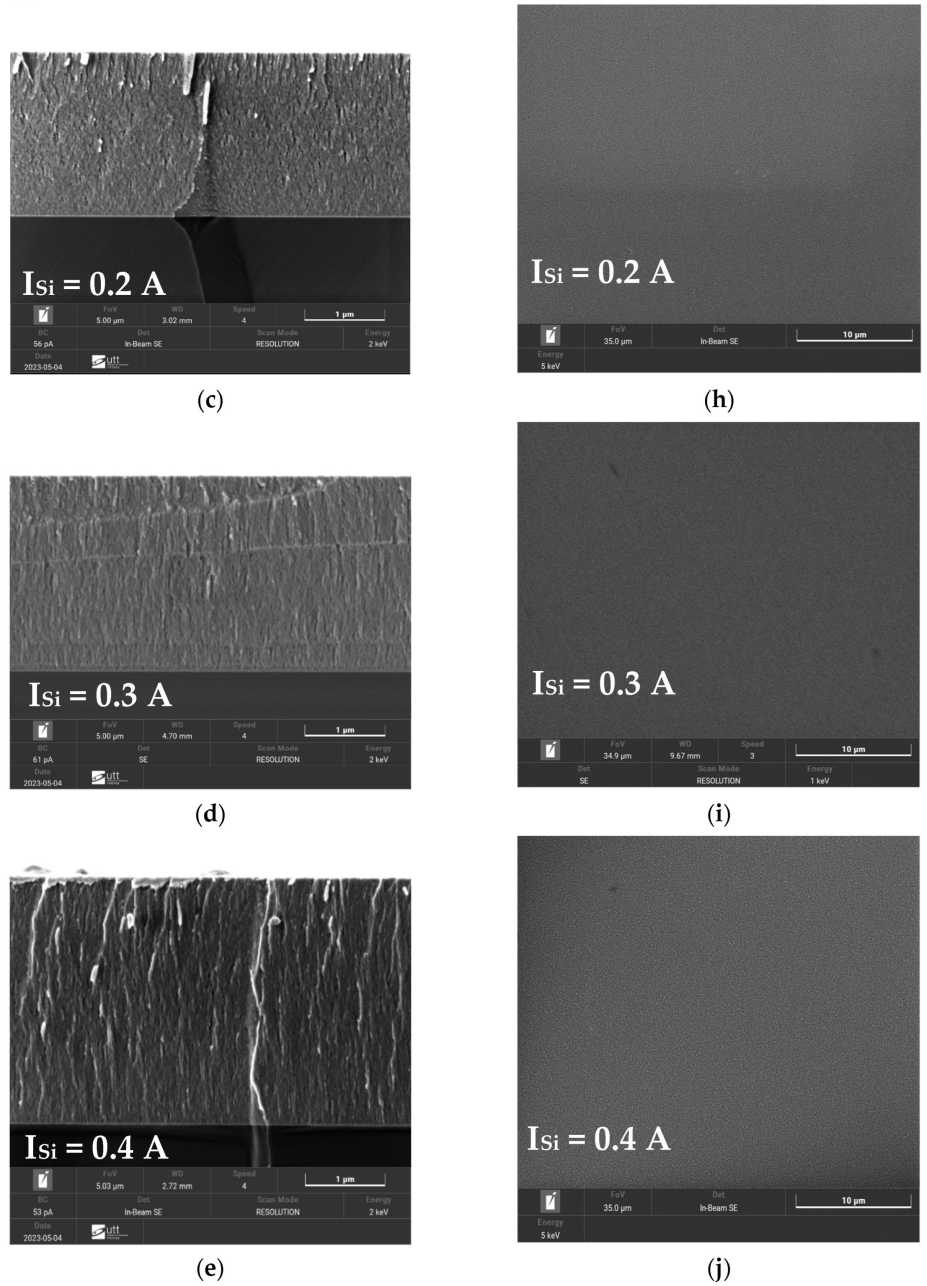
Cross-sectional (left images) and surface SEM morphologies (right images) of (AlTiZrHfTa)N*_x_*/SiN*_x_* RHECs for R_N2_ = 10% obtained for (**a**,**f**) I_Si_ = 0 A, (**b**,**g**) I_Si_ = 0.1 A, (**c**,**h**) I_Si_ = 0.2 A, (**d**,**i**) I_Si_ = 0.3 A, and (**e**,**j**) I_Si_ = 0.4 A.

The cross-sectional and top view SEM micrographs of the FCC (AlTiZrHfTa)N*_x_*/a-SiN*_x_* RHECs with various I_Si_ are shown in Figure 5. As we can notice from the figure, the coatings have a good combination with the silicon substrate. No obvious defects have been observed.

For the (AlTiZrHfTa)N*_x_* nitride coating (Figure 5a), large columnar morphology throughout the coating is observed, while the surface presents a pyramid-like aspect (Figure 5f). However, when FCC (AlTiZrHfTa)N*_x_*/a-SiN*_x_* is deposited, the coatings exhibit a dense, smooth cross-sectional morphology and no obvious columnar growth (Figure 5b–e). This aspect is linked to the formed nano-layered architecture, illustrated in Figure 2b, during the coating growth. As illustrated in Table 1 above, the calculated mean grain size decreases with the introduction of Si. This trend reflects the grain growth inhibition by the amorphous SiN*_x_* nano-layer [8,57,58].

### 3.4. Chemical Composition

**Figure 6:**
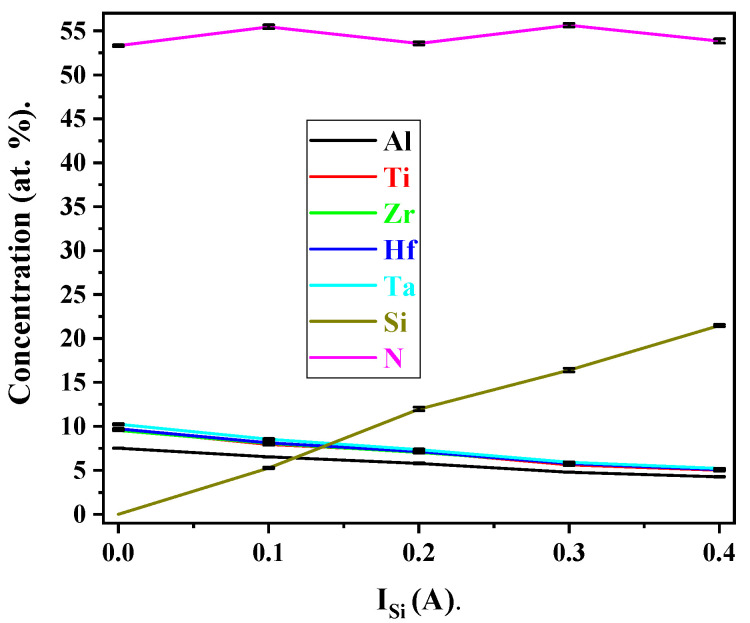
EPMA average element contents for FCC (AlTiZrHfTa)N*_x_*/a-SiN*_x_* coatings deposited at R_N2_ = 10% as a function of I_Si_.

Figure 6 presents the EPMA-detected global composition of (AlTiZrHfTa)N*_x_*/SiN*_x_* RHECs deposited at R_N2_ = 10%. The increase in silicon percentage in RHECs along with the increase in I_Si_ suggest that the newly added element may be effectively incorporated into the nitride layer as expected.

The chemical composition of the (AlTiZrHfTa)N*_x_* coating is as follows: Al = 7.5 at.%, Ti = 9.7 at.%, Zr = 9.5 at.%, Hf = 9.7 at.%, Ta = 10.3 at.%, and N = 53.3 at.%. The amount of Al was shown to be low in comparison to the target content (20 at.%). This phenomenon was explained in reference [42]. The composition stabilization of N content in the high-entropy nitride at R_N2_ = 10% is associated with the stabilization of the crystalline nitride solid solution and target poisoning [62].

As I_Si_ increased from 0.1 A to 0.4 A, the atomic percentage of Si in the FCC(AlTiZrHfTa)N*_x_*/a-SiN*_x_* deposited coatings raises from 5.3 at.% to 21.5 at.%, respectively (Table 2). Moreover, the contents of metals slightly decrease, resulting from the increase in silicon percentage and the formation of nitride structure.

### 3.5. Mechanical Properties

#### Hardness and Young’s Modulus of FCC(AlTiZrHfTa)N*_x_*/a-SiN*_x_* Thin Coatings

**Figure 7:**
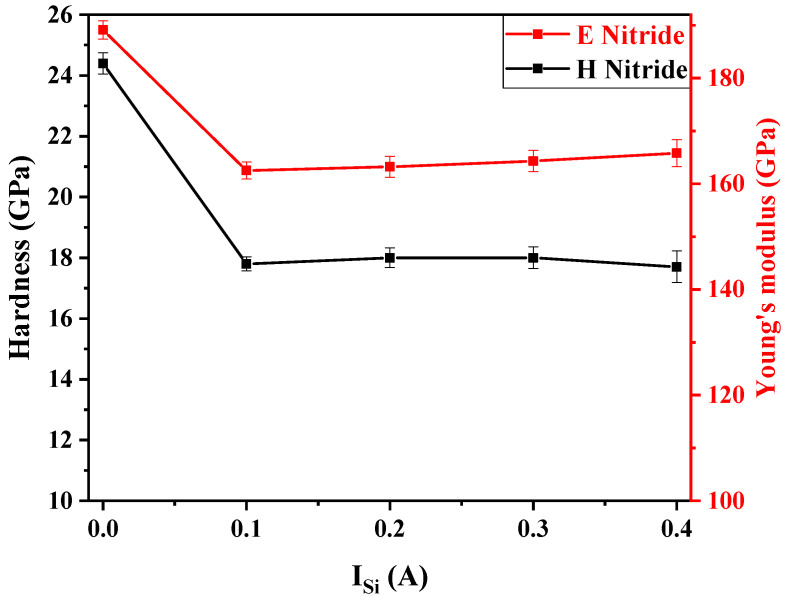
Hardness and Young’s modulus of (AlTiZrHfTa)N*_x_*/a-SiN*_x_*, R_N2_ = 10%, coatings at various I_Si_ (A).

Figure 7 depicts the evolution of hardness (H) and Young’s modulus (E) of FCC (AlTiZrHfTa)N*_x_*/a-SiN*_x_* (R_N2_ = 10%) coatings as a function of I_Si_. H decreased from 24.4 ± 0.3 GPa to 17.7 ± 0.5 GPa, while the Young’s modulus also decreased from 189.0 ± 1.7 GPa to around 162.5 ± 1.6 GPa. This is due to the formation of a nano-layered architecture (amorphous SiN*_x_* nano-layers and FCC crystalline (AlTiZrHfTa)N*_x_* nano-layers). The amorphous nano-layer (SiN*_x_*) hinders the growth of (AlTiZrHfTa)N*_x_* crystallites, leading to a sudden orientation drop as revealed in XRD patterns (cf. Figure 4) [63]. 

In general, nano-layered architecture leads to the hardness enhancement of the coating due to the blocking of dislocation movements at interfaces [57]. For example TiN/Si_3_N_4_ multilayers exhibited a hardness enhancement with Si_3_N_4_ layer thickness less than 1 nm (about 0.5 or 0.7 nm) [64,65]. However, Dong et al. [66] reported a change in hardness trend as a function of Si_3_N_4_ layer thickness for ZrN/Si_3_N_4_ nano-layered coating. Indeed, when the Si_3_N_4_ layer thickness is about 0.6 nm, the hardness is increased, due to the formation of a crystallized Si_3_N_4_ nano-layer forming coherent interfaces with the ZrN layer. However, when the thickness exceeds 1.1 nm, an amorphous growth of Si_3_N_4_ is observed, resulting in a significant hardness decrease. In our study we can consider that the non-isostructural nano-layered coating associated with the amorphous SiN_x_ with a thickness of 1.5 nm leads to a decline of mechanical properties. Similar trends have been observed for nano-layered coatings like Cr_2_N/Si_3_N_4_ [67], HfN/Si_3_N_4_ [68], and NbN/Si_3_N_4_ [69].

### 3.6. High-Temperature Oxidation Property

#### 3.6.1. Oxidation Resistance of FCC(AlTiZrHfTa)N*_x_*/a-SiN*_x_* Coatings

**Figure 8:**
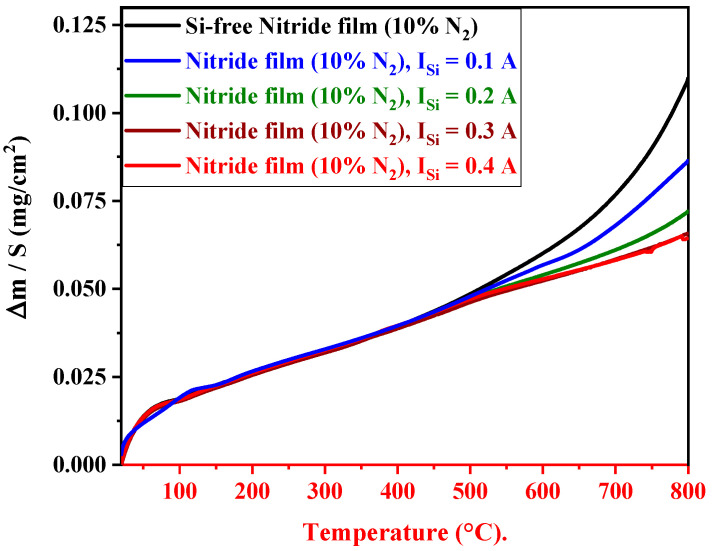
Weight gain per unit surface area ($\frac{\Delta m}{S},\;\frac{\mathrm{mg}}{{\mathrm{cm}}^{2}}$) as a function of temperature (°C) for FCC (AlTiZrHfTa)N and FCC (AlTiZrHfTa)N*_x_*/a-SiN*_x_* RHECs at R_N2_ = 10% obtained for various I_Si_.

Figure 8 depicts the dynamic thermogravimetric analysis (TGA) curves of the FCC (AlTiZrHfTa)N*_x_* and FCC (AlTiZrHfTa)N*_x_*/a-SiN*_x_* coatings from room temperature (RT) to 800 °C. The weight gain of the FCC (AlTiZrHfTa)N*_x_* coating (Figure 8) grows steadily between RT and 694 °C, followed by a rapid growth at higher temperatures. When the FCC (AlTiZrHfTa)N*_x_*/a-SiN*_x_* RHECs are deposited, the weight gain shows a drastic growth only after around 703 °C (for the RHECs obtained for I_Si_ = 0.1 A) but is largely lower than that of the FCC (AlTiZrHfTa)N*_x_* coating. When the Si content increases further, the critical oxidation temperature exceeds 800 °C. 

#### 3.6.2. Oxidation Kinetics of FCC(AlTiZrHfTa)N*_x_*/a-SiN*_x_* Coatings

**Figure 9:**
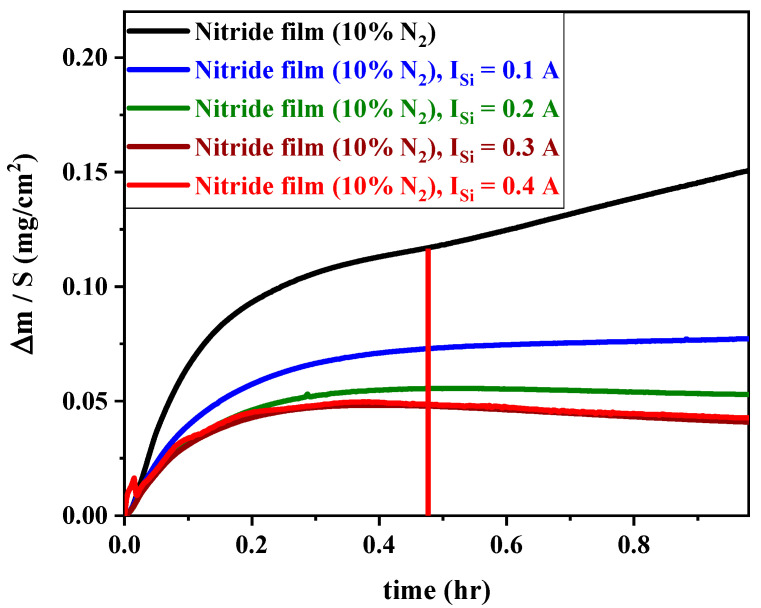
Oxidation kinetic curves FCC (AlTiZrHfTa)N/a-SiN*_x_* coatings at R_N2_ = 10% obtained for various I_Si_ at 800 °C (The vertical red line delimits the ending of parabolic growth and the beginning of the linear growth for the nitride film, black curve).

Figure 9 illustrates the isothermal thermogravimetric curves plotted during 1 h of exposure at 800 °C for FCC (AlTiZrHfTa)N*_x_*/a-SiN*_x_* coatings obtained for various I_Si_.

In the case of (AlTiZrHfTa)N*_x_* nitride coatings, there is an initial slower parabolic weight gain growth up to 0.112 mg/cm^2^, followed by a quick linear increase after around 28 min of oxidation. The linear rate law following by the parabolic growth (Figure 9) is related to breakaway oxidation effect. The linear weight gain is assumed to represent a change in the scale structure by formation of thick, porous oxide scale promoting a significant access of gaseous species towards the coating phase [70]. Gorr et al. [71] noticed a similar variation for the NbMoCrTiAl-1Si arc melted HEA.

When FCC (AlTiZrHfTa)N*_x_*/a-SiN*_x_* is deposited, the weight gain is drastically decreased. In addition, the breakaway point completely disappeared, which could be explained by the protectiveness enhancement of the new formed oxide [70,72].

##### Structure Analysis of Oxidized FCC(AlTiZrHfTa)N*_x_*/a-SiN*_x_* Coatings

**Figure 10:**
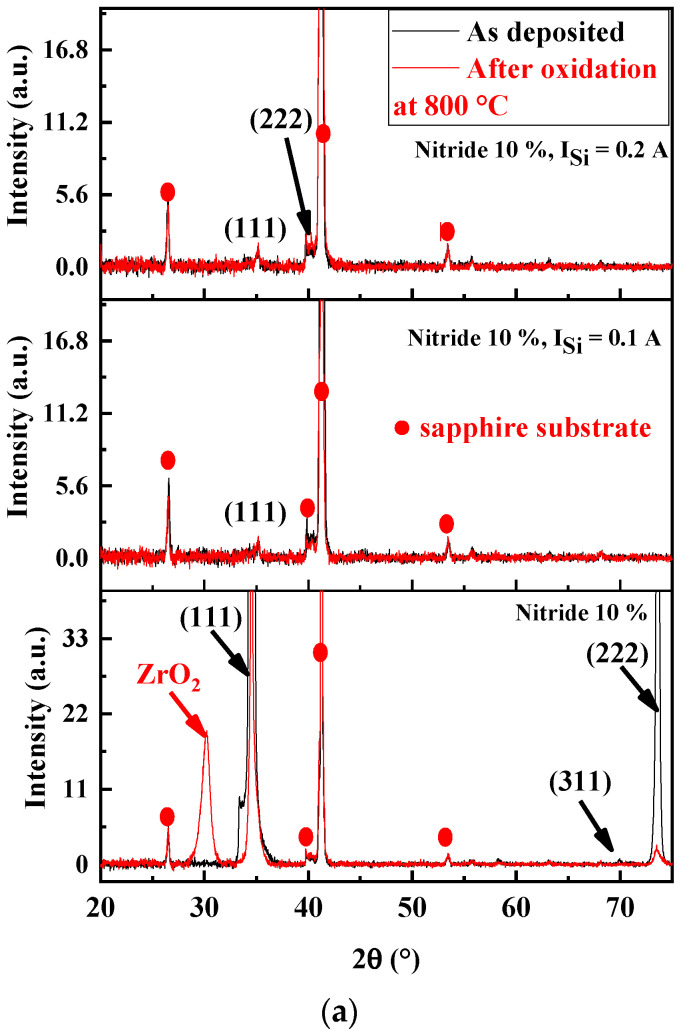

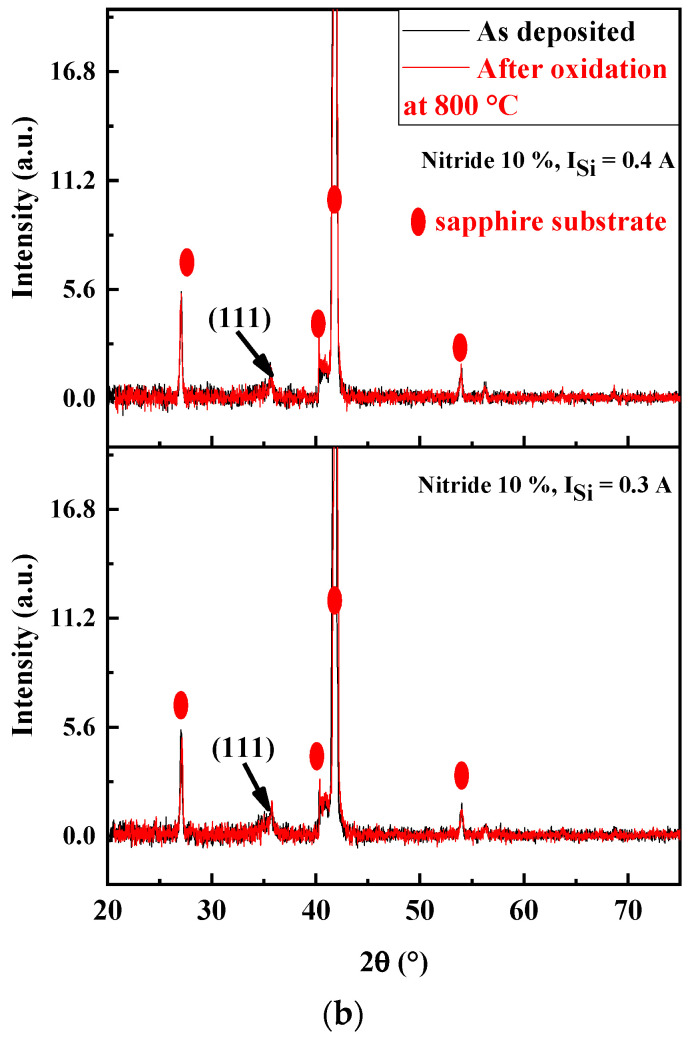
X-ray diffraction patterns of (**a**) (AlTiZrHfTa)N*_x_* nitride coatings and FCC (AlTiZrHfTa)N*_x_*/a-SiN*_x_* coatings obtained for I = 0.1 and 0.2 A. (**b**) FCC (AlTiZrHfTa)N*_x_*/a-SiN*_x_* coatings obtained for I = 0.3 and 0.4 A before and after 1 h of oxidation at 800 °C in a dry-air atmosphere.

Figure 10 presents the XRD patterns of (AlTiZrHfTa)N*_x_*/a-SiN*_x_* RHECs before and after 1 h of isothermal oxidation at 800 °C in a dry-air atmosphere. After oxidation, we noted a presence of a peak at 2 ≈ 30°, which could be identified as zirconia (ZrO_2_). In addition, a broadening of (111) and (222) peaks occur after oxidation. This broadening could be attributed to the formation of oxides [55,73]. For the (AlTiZrHfTa)N*_x_*/a-SiN*_x_* RHECs, no significant changes have been observed on XRD patterns whatever the current intensity on the Si target. At this stage, this analysis is not able to identify the oxide nature (complementary TEM and SEM results are presented below). It should be noted that neither peeling nor coating removal was observed on the annealed samples.

##### Morphology Analysis of Oxidized FCC(AlTiZrHfTa)N*_x_*/a-SiN*_x_* Coatings

**Figure 11:**
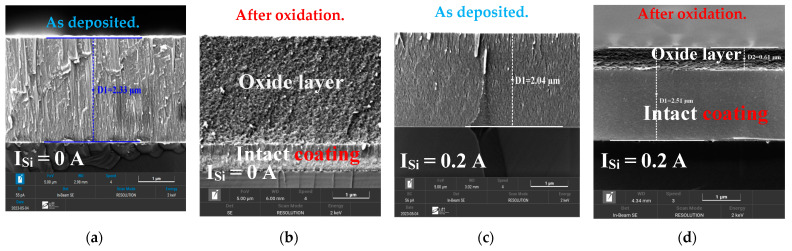

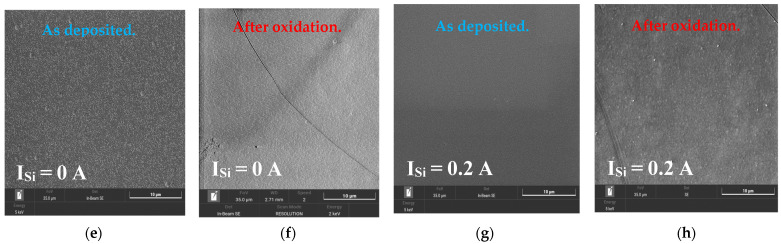
Cross-sectional and surface SEM micrographs of (AlTiZrHfTa)N(AlTiZrHfTa)N*_x_* RHECs (**a**,**e**) as deposited and (**b**,**f**) after 1 h oxidation at 800 °C, and FCC (AlTiZrHfTa)N/a-SiN*_x_* RHECs obtained for I_Si_ = 0.2 A (**c**,**g**) as deposited and (**d**,**h**) after 1 h oxidation at 800 °C.

To better understand of the evolution of the morphology of (AlTiZrHfTa)N*_x_*/a-SiN*_x_* RHECs following the oxidation process, SEM was used to investigate (AlTiZrHfTa)N*_x_* and FCC (AlTiZrHfTa)N*_x_*/a-SiN*_x_* obtained for I_Si_ = 0.2 A. Figure 11 shows their surface and cross-sectional morphologies, after 1 h of isothermal oxidation at 800 °C in a dry-air atmosphere. Figure 11f,h clearly illustrates the presence of cracks and pores on the coating’s surfaces. In the case of the FCC (AlTiZrHfTa)N*_x_*/a-SiN*_x_* coatings, obtained for I_Si_ = 0.2 A, the oxide layer is thinner, which indicates a better oxidation resistance compared to (AlTiZrHfTa)N*_x_* coatings (cf. Figure 9).

##### Microstructure Investigation of Oxidized FCC(AlTiZrHfTa)N*_x_*/a-SiN*_x_* RHECs

**Figure 12:**
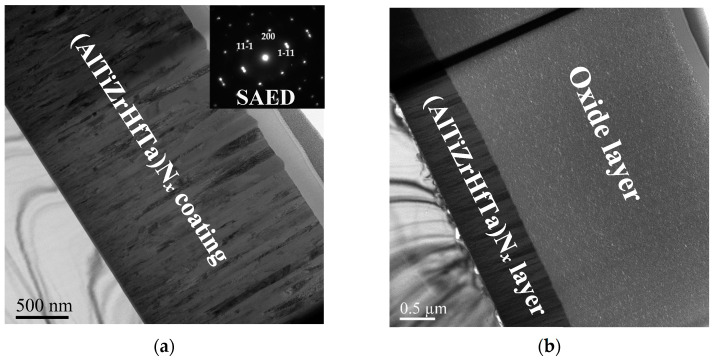

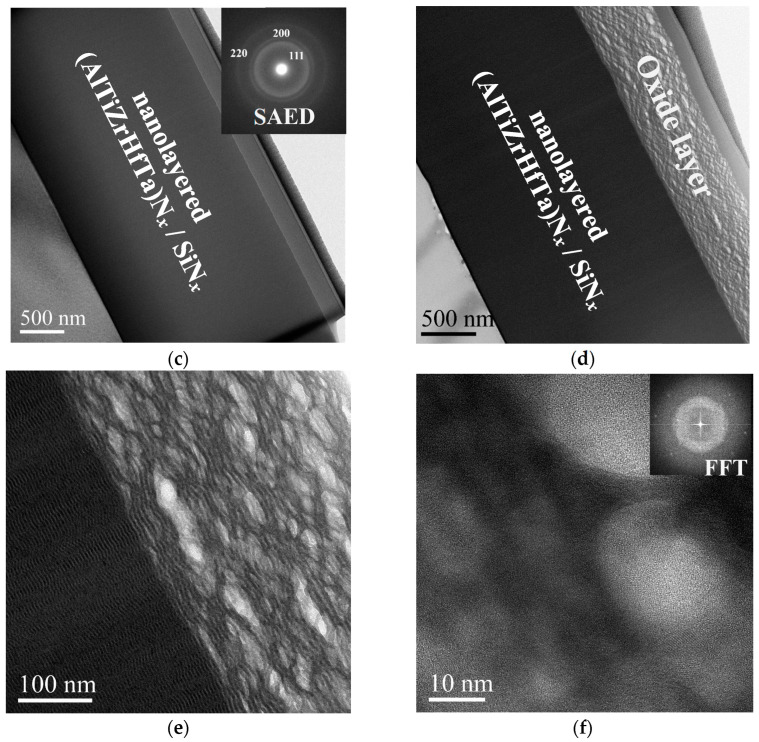
(**a**) Bright field TEM micrograph of intact nitride coating obtained with R_N2_ = 10% with associated SAED pattern. (**b**) Bright field TEM micrograph of the same coating after 1 h of oxidation at 800 °C. (**c**) Bright field TEM micrograph of intact FCC (AlTiZrHfTa)N_x_/a-Si_3_N*_x_* coating obtained for I_Si_ = 0.2 A with associated SAED pattern. (**d**) Bright field TEM micrograph of the FCC (AlTiZrHfTa)N*_x_*/a-SiN*_x_* coating obtained for I_Si_ = 0.2 A after 1 h of oxidation at 800 °C. (**e**) Zoom-in on oxidized layer of FCC (AlTiZrHfTa)N*_x_*/a-SiN*_x_* coating obtained for I_Si_ = 0.2 A coating. (**f**) HRTEM and associated FFT of oxidized zone of FCC (AlTiZrHfTa)N*_x_*/a-SiN*_x_* coating obtained for I_Si_ = 0.2 A.

The cross-sectional microstructure of oxidized (AlTiZrHfTa)N*_x_* RHECs at R_N2_ = 10% and the (AlTiZrHfTa)N*_x_*/a-SiN*_x_* coating obtained for I_Si_ = 0.2 A was analyzed by using TEM (Figure 12). The as-deposited (AlTiZrHfTa)N*_x_* nitride coating exhibits a columnar growth (Figure 12a) with (111) preferred orientation as shown by the inset SAED pattern. After oxidation, the (AlTiZrHfTa)N*_x_* nitride coating reveals two separate zones: an intact coating with black contrast at the bottom and a homogeneous thick (≈2.5 μm, Figure 11b) and porous oxide layer on the top (depicted in reference [42]). For FCC (AlTiZrHfTa)N*_x_*/a-SiN*_x_* coating, obtained for I_Si_ = 0.2 A, a nano-layered architecture is observed (cf. Figure 2b). After oxidation of the (AlTiZrHfTa)N*_x_*/SiN*_x_* coating, two different zones are observed as well: intact coating with a nano-layered architecture at the bottom (Figure 12e) and a homogeneous thin (≈600 nm) and porous oxide layer on the top (Figure 12d,e).

HRTEM image of the oxide of the (AlTiZrHfTa)N*_x_*/SiN*_x_* coating, obtained for I_Si_ = 0.2 A, is presented in Figure 12f. The oxide layer exhibits an amorphous aspect. This is further verified by the corresponding FFT pattern, which shows a circular diffuse ring (Figure 11f).

**Figure 13:**
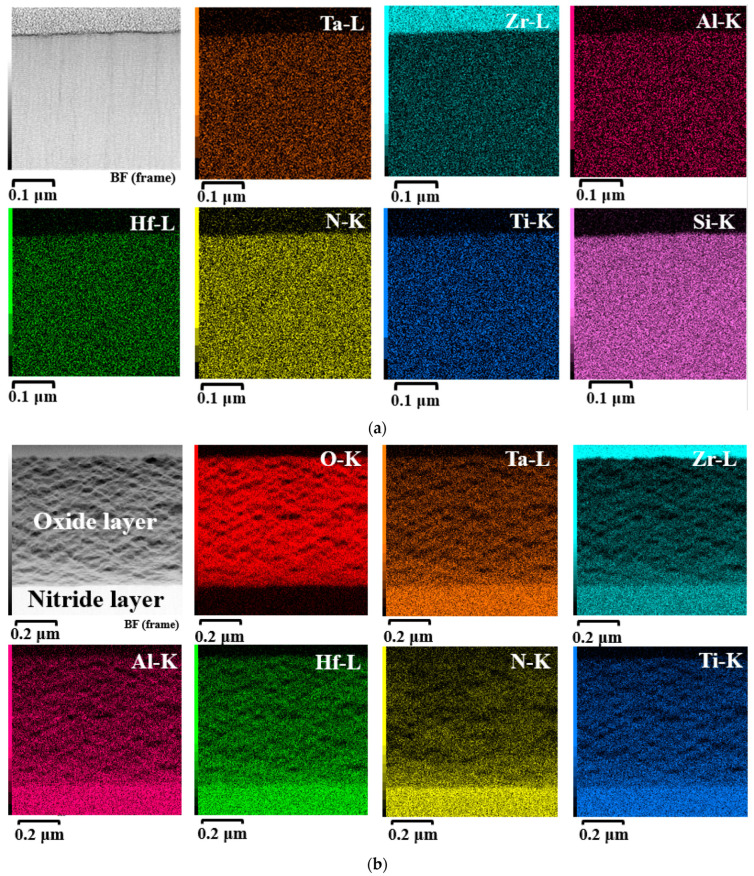
STEM EDS mapping of the FCC (AlTiZrHfTa)N*_x_*/a-SiN*_x_* coating obtained for I_Si_ = 0.2 A: (**a**) sections before and (**b**) zoom-in on oxidized zone after 1 h ofoxidation at 800 °C.

STEM-EDS mapping of the FCC (AlTiZrHfTa)N*_x_*/a-SiN*_x_* coating obtained for I_Si_ = 0.2 A, before and after the oxidation process on the oxidized zone area, was performed. The results are presented in the (Figure 13). 

After 1 h of oxidation at 800 °C, we found a uniform distribution of metallic elements throughout the (AlTiZrHfTa)N*_x_*/SiN*_x_* RHECs and oxide layer (Figure 13). As a result, a mixed oxide is formed [74]. It should be noted that the amorphous aspect of the oxide layer has also been observed for the (AlTiZrHfTa)N*_x_* nitride coating [42].

##### Oxidation Rate through k_p_ Analysis

**Figure 14:**
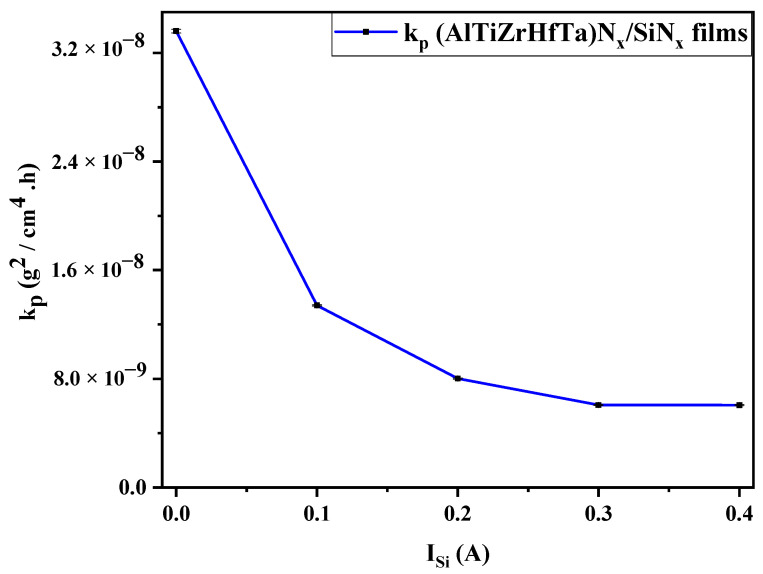
Kinetic constant k_p_ of FCC (AlTiZrHfTa)N*_x_*/a-SiN*_x_* nitride coatings obtained at (R_N2_ = 10%) after 1 h of oxidation at 800 °C as a function of I_Si_ (A).

Figure 14 traces the parabolic rate constant k_p_ g^2^ cm^−4^ h^−1^ at 800 °C for the investigated (AlTiZrHfTa)N*_x_* and (AlTiZrHfTa)N*_x_*/SiN*_x_* coatings. k_p_ is calculated according to (Equation (1)) [75]. For the (AlTiZrHfTa)N*_x_* coating, k_p_ is calculated at 3.36 × 10^−8^ g^2^ cm^−4^ h^−1^ for the sample tested at 800 °C. However, in the case of the (AlTiZrHfTa)N*_x_*/SiN*_x_* coating, obtained for I_Si_ = 0.2 A, the kinetic constant k_p_ decreased to 6.06 × 10^−9^ g^2^ cm^−4^ h^−1^ (Figure 14). This decreasing tendency sustains the previous oxidation kinetic curves’ evolution (cf. Figure 9), revealing an oxidation resistance enhancement of the (AlTiZrHfTa)N*_x_*/SiN*_x_* coating compared to the (AlTiZrHfTa)N*_x_* coating.

(1) $${\left(\frac{\Delta m}{S}\right)}^{2}=k_{p}\cdot t,$$

##### Activation Energy E_a_

The oxidation activation energy (E_a_) is of fundamental importance to understanding the oxidation mechanisms. It can be evaluated from the Arrhenius formula (Equation (2)) [76], by linear fitting of the kinetic constant (oxidation rate) at different oxidation temperatures (700 °C, 750 °C, and 800 °C):

(2) $$k_{p}=kp_{0}\;\exp\;(-\frac{E_{a}}{R\;T}),$$

R: molar gas constant, k_p0_: oxidation rate constant, and T: temperature. 

The activation energy of (AlTiZrHfTa)N*_x_* nitride coating was equal to 90.8 kJ·mol^−1^ [42]. However, the activation energy increases to a value of 126.52 kJ·mol^−1^ for the (AlTiZrHfTa)N*_x_*/SiN*_x_* coating obtained for I_Si_ = 0.2 A (Table 3). Even at this oxidation enhancement, the values remain low when compared to those of other alloys, as shown in Table 3.

#### 3.6.3. Discussion of the Oxidation Mechanisms

The present study explored the oxidation resistance of FCC (AlTiZrHfTa)N*_x_*/SiN*_x_* thin coatings. When Si is introduced, the oxidation resistance is drastically enhanced, according to various parameters such as the increase in oxidation temperature, breakaway disappearance, on weight gain curve, and k_p_ decreasing trend during 1 h of oxidation at 800 °C (cf. Figure 8, Figure 9 and Figure 14). SEM and TEM analyses illustrated the formation of a dense nano-layered architecture due to the sweeping mode during the deposition process. Moreover, XPS and XRD patterns depicted the presence of FCC (AlTiZrHfTa)N*_x_* and amorphous SiN*_x_* phases.

It should be mentioned that the amorphous (SiN*_x_*) layer is known for its elevated resistance to oxygen diffusion at high temperatures [56,66,79,80]. Moreover, the oxidation rate decrease could also be related to a relatively smaller residual stress throughout the coating, as a result of multiple interfaces between the formed nano-layers [36,81,82,83]. This phenomenon differs significantly from monolithically grown (AlTiZrHfTa)N*_x_*’s oxidation behavior (Figure 9 and Figure 11). In addition, the high number of interfaces of FCC (AlTiZrHfTa)N/a-SiN*_x_* nano-layers lead to a reduction in the interconnection of pores and defects penetrating through the coating that may result in oxygen diffusion into the oxidized coating [83].

**Figure 15:**
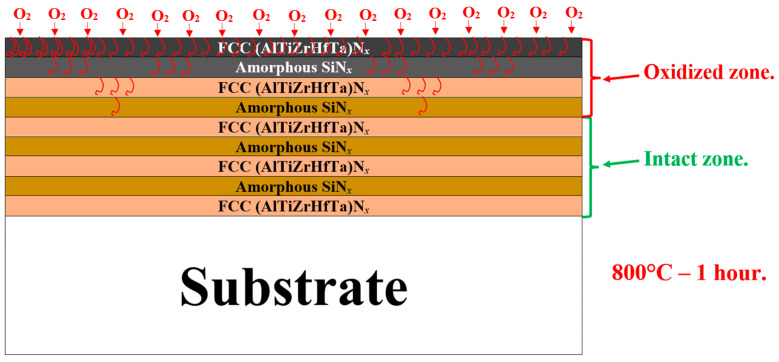
Schematic drawing, illustrating the oxidation behavior of the nitride coatings in the presence of Si.

A schematic model is proposed in Figure 15 to illustrate the SiN*_x_* inhibiting effect, when the (AlTiZrHfTa)N*_x_*/a-SiN*_x_* thin coating is exposed to oxygen. Because of their amorphous nature, SiN*_x_* nano-layers act as barrier layers (shields), which could inhibit the mutual diffusion of additional metallic atoms and decreases the oxidation reaction [36]. Steyer et.al [84] showed the oxidation resistance enhancement of TiN coating by the segregation of amorphous SiN_x_ at the grain boundaries, leading to an increase in the protective shielding effect. The results reported in this study verified that a coating synthesized by the current alloy design efficiently slowed the oxidation rate.

## 4. Conclusions

(AlTiZrHfTa)N*_x_*/SiN*_x_* RHECs were synthesized by DCMS in two nitrogen ratios (R_N2_ = 0% and 10%).

The effect of Si’s addition on the structure, microstructure, mechanical properties, and oxidation behavior were investigated. The current study is mainly focused on the oxidation resistance enhancement of these so-called “refractory” HEA coatings by using an alloying approach and promoting nano-layered architecture.

- The deposition of nano-layered FCC (AlTiZrHfTa)N*_x_*/a-SiN*_x_* coatings results in a density increase in the nitride coatings.
- The deposition of the nano-layered FCC (AlTiZrHfTa)N*_x_*/a-SiN*_x_* coatings leads to the decrease in hardness and Young’s modulus up to H = 17.7 ± 0.5 GPa and E = 162.5 ± 1.6 GPa. The softening of the coatings results from the formation of the amorphous SiN*_x_* nano-layers, hindering the growth of the FCC (AlTiZrHfTa)N*_x_* nano-layers.
- The deposition of the nano-layered FCC (AlTiZrHfTa)N*_x_*/a-SiN*_x_* coating improved the oxidation resistance at 800 °C. The increase in I_Si_ significantly decreased the parabolic rate constant k_p_ from 3.36 × 10^−8^ g^2^ cm^−4^ h^−1^ for FCC (AlTiZrHfTa)N*_x_* coating to 6.06 × 10^−9^ g^2^ cm^−4^ h^−1^ for FCC (AlTiZrHfTa)N*_x_*/a-SiN*_x_* coatings at 800 °C.
- The activation energy E_a_ has increased from 90.8 kJ·mol^−1^ for the FCC (AlTiZrHfTa)N*_x_* coating to 126.52 kJ·mol^−1^ for the FCC (AlTiZrHfTa)N*_x_*/a-SiN*_x_* coating obtained for I_Si_ = 0.2 A. This trend reflects an oxidation resistance improvement due to the formation of the amorphous SiN*_x_* nano-layer in alternance with FCC (AlTiZrHfTa)N*_x_*.

The deposition of FCC (AlTiZrHfTa)N*_x_*/a-SiN*_x_* results in the formation of an inhibiting amorphous SiN*_x_* nano-layer, protecting FCC (AlTiZrHfTa)N*_x_* crystallites from oxygen onslaught, thus improving their oxidation resistance.

The results obtained in this study illustrate the effectiveness of using an alloying approach to further enhance the RHEAs’ efficiency, particularly toward high-temperature applications.

## Figures and Tables

**Table 1:** The calculated mean grain size values, measured by the Scherrer equation, of the FCC (AlTiZrHfTa)N/a-SiN*_x_* coatings as a function of I_Si_.

Coating	I_Si_ (A)	Average Grain Size, Ø (nm)
(AlTiZrHfTa)N (R_N2_ = 10%)	0	45.92
(AlTiZrHfTa)N*_x_*/SiN*_x_* (R_N2_ = 10%)	0.1	2.1
(AlTiZrHfTa)N*_x_*/SiN*_x_* (R_N2_ = 10%)	0.2	2
(AlTiZrHfTa)N*_x_*/SiN*_x_* (R_N2_ = 10%)	0.3	2
(AlTiZrHfTa)N*_x_*/SiN*_x_* (R_N2_ = 10%)	0.4	2.2

**Table 2:** Applied current on Si target I_Si_ (A) and associated global Si percentage (at.%) into the FCC (AlTiZrHfTa)-N/a-SiN*_x_* coatings.

I_Si_ (A)	Corresponding Si Atomic Percentage (at.%)
0	0
0.1	5.3
0.2	12
0.3	16.4
0.4	21.5

**Table 3:** Activation energy E_a_ of Si-free nitride coating and Si-doped (AlTiZrHfTa)N*_x_*/a-SiN*_x_* coating obtained for I_Si_ = 0.2 A, and different investigated thin coatings [42,77,78].

Coating	E_a_ (Activation Energy) (kJ·mol^−1^)	E_a_ Error	Reference
(AlTiZrHfTa)N*_x_* (R_N2_ = 10%)	90.8	0.05	[42]
(AlTiZrHfTa)N*_x_*/SiN*_x_* (R_N2_ = 10%, I_Si_ = 0.2 A)	126.52	0.02	This study
CrN	243	-	[77]
CrAlN	280	-	[77]
TiN	193	-	[77]
TiSiN	260	-	[77]
(AlCrTaTiZr) N	208.6	-	[78]

## Data Availability

The raw data supporting the conclusions of this article will be made available by the authors on request.
